# Swirl Flow Bioreactor coupled with Cu-alginate beads: A system for the eradication of Coliform and *Escherichia coli* from biological effluents

**DOI:** 10.1038/srep09461

**Published:** 2015-05-21

**Authors:** Sov Atkinson, Simon F. Thomas, Paul Goddard, Rachel M. Bransgrove, Paul T. Mason, Ajeet Oak, Anand Bansode, Rohit Patankar, Zachary D. Gleason, Marissa K. Sim, Andrew Whitesell, Michael J. Allen

**Affiliations:** 1 Plymouth Marine Laboratory, Prospect Place, The Hoe, Plymouth, PL1 3DH, UK; 2 PML Applications, Prospect Place, The Hoe, Plymouth, PL1 3DH, UK; 3 Protein Technologies Ltd, Williams House, Lloyd St North, Manchester M15 6SE, UK; 4 PriMove Infrastructure Development Consultants Pvt. Ltd, Paud Road, Pune, 411 038, India; 5 The Sewage Treatment Plant, Rajiv Gandhi Infotech Park, Hinjewadi, Pune, Maharashtra, India; 6 Cascade Designs, Inc. 4000 First Avenue South, Seattle, Washington, 98134, USA; 7 Beaumont Design, 232 Pouli Road, Kailua, Hawaii, 96734, USA

## Abstract

It is estimated that approximately 1.1 billion people globally drink unsafe water. We previously reported both a novel copper-alginate bead, which quickly reduces pathogen loading in waste streams and the incorporation of these beads into a novel swirl flow bioreactor (SFB), of low capital and running costs and of simple construction from commercially available plumbing pipes and fittings. The purpose of the present study was to trial this system for pathogen reduction in waste streams from an operating Dewats system in Hinjewadi, Pune, India and in both simulated and real waste streams in Seattle, Washington, USA. The trials in India, showed a complete inactivation of coliforms in the discharged effluent (Mean Log removal Value (MLRV) = 3.51), accompanied by a total inactivation of *E. coli* with a MLRV of 1.95. The secondary clarifier effluent also showed a 4.38 MLRV in viable coliforms during treatment. However, the system was slightly less effective in reducing *E. coli* viability, with a MLRV of 1.80. The trials in Seattle also demonstrated the efficacy of the system in the reduction of viable bacteria, with a LRV of 5.67 observed of viable *Raoultella terrigena* cells (100%).

Human development and rapid population growth exert numerous pressures on the quality of and access to water resources. This is felt strongest at the interface between water and human health; where infectious, waterborne diseases remain the leading causes of human morbidity and mortality worldwide. It is estimated that approximately 1.1 billion people globally drink unsafe water^1^. The World Bank estimates 21% of the communicable diseases, in India, are water related. Of these diseases, diarrhoea alone is estimated to have killed over 535,000 Indians in 2004^2^. The highest mortality from diarrhoea is in children under the age of five, highlighting an urgent need for focused interventions to prevent diarrhoeal disease in this age group^2^. The cost of diarrhoeal disease-associated morbidity is vast, amounting to an estimated $US3.33 per household for every episode^3^. Thus, the subsequent socio-economic effect of the contamination of potable water supplies, in many developing regions, is a significant contribution to the continuation of poor living standards. The water supply of a typical rural village in a developing country is prone to faecal contamination, due to the close proximity of the water supply to farmed areas and sanitation facilities^4^. In contrast to large urban cities, in developing countries, the major source of water pollution is the discharge of untreated domestic wastewater into the watercourse^4^, which contain microorganisms of intestinal origin, such as *helminth ova* and faecal coliform bacteria. Although the effects of poor sanitation in rural areas of developing countries are well characterised^1^,^2^,^3^,^4^,^5^, a potential larger problem exists in peri-urban areas which are currently expanding around the edges of existing conurbations^6^. Such areas often have no or poor planning, and are typically occupied by poorer communities, either migrating from rural locales or displaced from more expensive urban locations^7^,^8^. Often such areas have no access to centralised wastewater treatment and the cost of the extension of existing provisions is prohibitive^5^,^6^,^7^, therefore the innovative use of existing and novel strategies for the decentralised treatment of waste are required, often on a site-specific basis^7^,^9^,^10^,^11^. This is the essence of the Decentralized Wastewater Treatment Systems (Dewats) strategy^5^,^7^,^8^.

A typical Dewats system consists of primary and secondary treatment, and disposal (or utilisation) of solids and treated water^12^. The primary treatment may be as simple as a septic tank, to remove settleable solids (and provide limited anaerobic treatment), which can be used in areas of poor soil and high groundwater^12^,^13^. Modifications of the above system which enable aerobic treatment of the effluent and prevent floating solids from entering the secondary treatment^12^. Although cheap and requiring little maintenance, they are prone to failure^14^,^15^ and even when operating effectively may still leave a pathogen-rich waste stream^4^.

Secondary treatment options, based on sand filters, provide effective removal of pathogens in areas with deep permeable soils, but are ineffective in other locales with highly permeable soil type^15^. Other solutions, such as facultative and aerated lagoons, and constructed wetlands, provide effective removal of pathogens, but require extensive land areas and provide a serious public health risk in areas where malaria, Dengue fever, yellow fever, Hepatitis A and Cholera are endemic^11^. For a comprehensive review of existing treatment options, consult Massoud *et al*^11^ and Parkinson and Taylor^13^.

Therefore a requirement still exists, in many locations, for an effective pathogen destruction technology for incorporation into Dewats systems. We have described previously both a novel copper-alginate bead, which quickly reduces pathogen loading in waste streams^16^ and the incorporation of these beads into a novel swirl flow reactor, a system of low capital and running costs and of simple construction from commercially available plumbing pipes and fittings^17^. In the course of this paper we describe the trialling of this system for pathogen reduction in waste streams from an operating Dewats system in Hinjewadi, Pune, India and in both simulated and real waste streams in Seattle, Washington, USA.

## Results

### Chemical and physical parameters of waste streams

**Figure 1:**
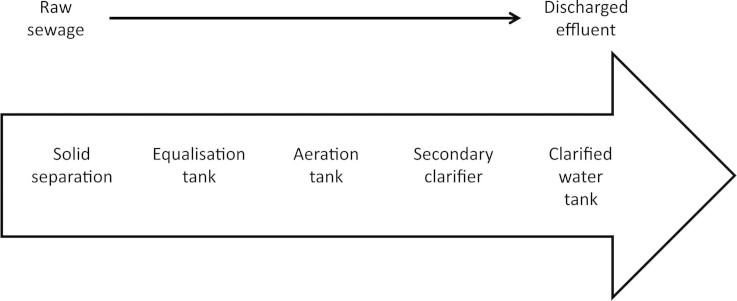
Process flow diagram for Hinjewadi sewerage treatment plant.

The Hinjewadi Dewats system consisted of a coarse solid separator, leading to an equalisation tank with subsequent aerobic treatment. The water was then further treated in a secondary clarifier, sent to a storage tank prior to discharge (Figure 1). The resulting effluent was of clean appearance with a pH of 7.2, and of a low suspended solid content and Chemical Oxygen Demand (COD) at 55 and 9 mg/L respectively (Table 1). The effluent from the secondary clarifier still contained some obvious suspended solids and brown discolouration (Table 1). The pH was slightly lower than the discharged effluent at 7.1 and the TSS and COD were 141 and 84 mg/L, respectively (Table 1); whereas, the effluent from the aeration tank was of a higher suspended solid (132 mg/L) and was of a dark brown colouration (Table 1). The equalisation and aeration tank produced a 54.4% and 36.4% reduction in COD and TSS, respectively, whilst the secondary clarifier produced a further reduction of 61% in COD and 89.3% in TSS.

The NSF P231 challenge water was prepared by Cascade Design Inc., from a prescribed recipe contained 28.57 mg/L of humic acids, a COD of 44.8 mg/L, and so showed some discolouration, and a known concentration (100 mg/L) of suspended solids was added. Both the secondary effluent and the septic tank effluent from the Seattle trial were of far higher COD (1342 and 11,040 mg/L) than the other effluents, and had much higher suspended solid contents (4600 and 251,950 mg/L [Table 1]).

### Field trial results

There was a reduction in CFU/mL of both total coliforms and *E. coli* during the initial tests of the swirl flow bioreactor running without beads on the discharged effluent from Hinjewadi, with log removal values (LRV) of 0.26 (±0.13) and 0.18 (±0.09) respectively (Table 2). However, this effect was not repeated when the secondary clarifier effluent was tested, in fact a small increase in coliform numbers was observed, accompanied by an LRV of 0.249 (±0.12) for *E. coli* (Table 2).

The complete inactivation of coliforms was observed for both runs with the discharged effluent when the swirl flow bioreactor was loaded with Cu-alginate beads (LRV = 3.12 [±1.57] and 3.90 [±1.95]), accompanied by a total inactivation of *E. coli* in the first run (LRV = 2.31 [±1.16]) and a 97.89% reduction in viable cells during the second run (LRV = 1.59 [±0.79]). The secondary clarifier effluent also showed a 4.16 (±2.04) and 4.59 (±2.14) LRV in viable coliforms during treatment. However, the system was slightly less effective in reducing *E. coli* viability, with a reduction of 95.82 and 99.34% (LRV = 1.40 [±0.70] and 2.19 [±1.09]) observed during the trials (Table 2).

Using the higher TSS containing aeration tank effluent, a 4.86 (±2.43) LRV of viable coliforms was observed, accompanied with a LRV in viable *E. coli* cells of 1.54, ± 0.77 (97.11% [Table 2]). The bead integrity was adversely affected by this waste stream, resulting in the presence of bead fragments in the system. These fragments settled out within 20 seconds post agitation.

The trials in Seattle also demonstrated the efficacy of the system in the reduction of viable bacteria, with a LRV of 5.67 observed of viable *Raoultella terrigena* cells (100%) using NSF P231 challenge water (Table 2). Treatment also resulted in total loss of viability of *E. coli* cells in secondary effluent with an LRV of 5.04. However, the system was much less effective in reducing pathogen viability in the higher TSS/COD effluent from a septic tank, with widely varying reductions in *E. coli* viability of between 99.47 and 37.55% (LRV = 2.27 and 0.20) observed (Table 2). Again, the beads showed signs of fragmentation during these trials, but the resulting particles were easily removed upon settling.

## Discussion

We have previously demonstrated that the Cu-alginate beads produced a synergistic antimicrobial effect^16^ and that these beads in conjunction with a novel swirl flow bioreactor can effectively treat larger volumes of water in a laboratory situation^17^. Here, we present the first field trials of this system performed in two separate continents. The system provided effective reduction of viable coliforms in waste streams containing high colour, COD and TSS in both Hinjewadi and Seattle and, in the reduction of viable *E. coli* and *R. terrigena* in Seattle, with LRV of up to 5.04 and 5.67 respectively. However, the reduction in viable *E. coli* cells during the Hinjewadi trials was consistently lower, indicating possible intrinsic resistance^18^, or more probably the presence of viable but non-culturable cells detected by the enzyme-based fluorescent assay^19^ used during the field study as compared to the traditional culture-based approached used by ourselves previously^16^ and during the Seattle-based trials. The recovery of these cells was not assessed during this study, but will be investigated at a later stage.

The limitations of the system are demonstrated by the inefficiency in pathogen reduction observed during the trial on the septic tank effluent, a “worst case” scenario, for which the system was not designed. The system is intended to offer a solution for secondary and tertiary effluent, which still has an unacceptable pathogen loading. These effluents are typically of lower suspended solids and with some residual colour and the use of this technology may supplement existing technologies, which offer effective solids removal.

This system still requires improvement and scale up before its worth can be definitively calculated. For example, the integrity and longevity of the beads requires continuing work, and bead integrity was adversely affected by the higher COD and TSS waste streams. This may be due to competing ions within the waste effecting the Ca-alginate or Cu-alginate interactions within the gel matrix^20^; these interactions are less resistant than chemical bonds and can cause alginate beads to swell and eventually dissolution. In addition, the effect of this system on reducing waterborne viruses, pathogenic zooplankton and *helminth* eggs and larvae is uncertain, as these diseases provide high morbidity and financial burden in addition to bacteria^21^,^22^. However, the system is cheap and easily constructed from locally available parts, and the moving parts consist of “off the shelf” components and items that can be replicated on a 3-D printer.

The Dewats approach to wastewater treatment represents a philosophy of combining existing and novel technologies on a site to site basis, whilst considering socio-economic, practical, geographic and even religious factors^5^,^6^,^10^,^23^, whilst offering the potential for reuse of the waste stream for irrigation^24^ and potentially, the solids as fuel^25^. The system we have trialled, shows considerable promise for reducing pathogen numbers in a variety of waste streams. The viability of the use of Cu-alginate within the swirl flow bioreactor is however questionable, as issues concerning integrity and the subsequent release of copper into the treated water have been identified. A more robust active agent may prove more amenable to integration into the system. A further developed swirl flow bioreactor could offer a viable alternative to UV sterilisation in waste streams that are typically resistant to this technology^26^,^27^,^28^ or in locales where geological conditions, or the prevalence of Mosquito-borne illness, prevent the use of soil filtration^15^, stabilisation ponds^13^ or constructed wetlands^11^.”

## Methods

### Sample Collection

**Figure 2:**
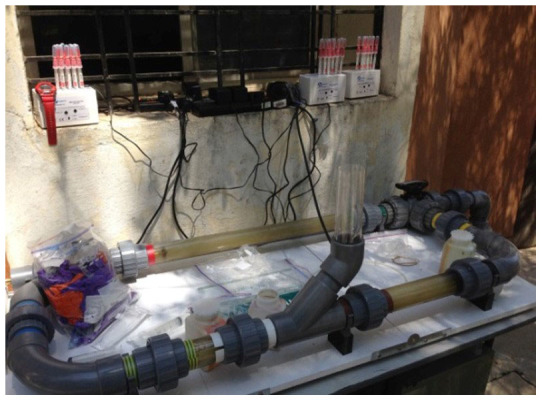
Swirl Flow Bioreactor set up in the field study at The Sewage Treatment Plant, Rajiv Gandhi Infotech Park, Hinjewadi, Pune, Maharashtra, India.

All field work in this study was carried out at The STP plant, Rajiv Gandhi Infotech Park, Hinjewadi, Pune, Maharashtra, India. Figure 2, shows the ad-hoc setup of the Swirl Flow Bioreactor and Figure 1 shows the process flow diagram of where each effluent originated and collected from. Effluents were obtained from the overflow of the aeration tank, overflow from the clarified water tank and the discharged water.

### Swirl Flow Bioreactor (in absence of Cu-alginate beads) Microbial Assay

**Figure 3:**
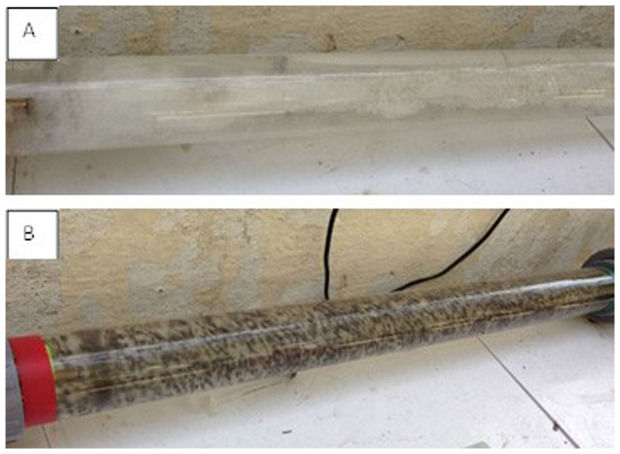
The turbulence and swirl characteristics of the swirl flow bioreactor, A) without beads and B) with beads.

Each effluent (~6.5 L) was decanted into the Swirl Flow Bioreactor (see Figure 2 and further detailed description of the Swirl Flow Bioreactor can be seen in Thomas *et al*^17^) and the bioreactor started without the presence of the Cu-Alginate composite beads (see Figure 3a). Triplicate samples were taken for microbial assay at 0, 10, 20 and 30 mins. Total ATP, *E. coli* and Coliform assay analyses were completed using fluorescence-based enzyme assays (Hygiena, Hertfordshire, UK), according to the manufacture's instruction and a handheld Luminometer (Model EnSURE, Hygiena, Hertfordshire, UK). The raw Relative Light Unit (RLU) readings from the commercialised detection kit were converted to CFU/mL according to the manufacturer's formula. The Swirl Flow Bioreactor was rinsed repeatedly with clean water after use.

### Effect of Cu-alginate composite beads on *E. coli* in effluents

A similar procedure to the study without Cu-alginate beads as above was followed. However, the Cu-alginate composite beads (350 g) were added to the effluent in the Swirl Flow Bioreactor (see Figure 3b) after the initial triplicate samples (at 0 minute) were removed. Further triplicate samples were taken at 10, 20 and 30 mins, for microbial assay using the Hygiena detection kit.

### Cu-alginate Beads Formation

Sodium alginate (Sigma Aldrich, Poole, UK) (4% w:v) was slowly dissolved, via stepwise addition into Milli-Q water (Veolia, High Wycombe, UK), whilst stirring on a magnetic stirrer at 300 rpm. Once dissolved, Cu microparticles (d50 = 3 μm) (FinePowder, USA) were added at a concentration of 4% (w:v). The particles were dispersed by stirring and vigorous shaking. The mixture was stored at 4°C until further processing. Beads were formed by the drop wise addition of alginate/copper mixture via a 0.8 mm gauge needle into a ~4°C solution of CaCl_2(aq)_ (2.5% w:v) with minimal stirring. Once formed, beads were stored at 4°C in the CaCl_2_ to harden overnight then stirred in CuSO_4 (aq)_ (~2% w:v) for 1 hour, washed thoroughly and stored in MilliQ water for further use.

### Chemical Oxygen Demand (COD) analysis

COD was analysed using the method outlined in the Standard Methods for the Examination of Water and Wastewater: 5220 Chemical Oxygen Demand (COD)^29^.

### Swirl Flow Bioreactor Microbial Assay Study at Cascade Designs Inc., Seattle, USA

Secondary effluent originated from a Municipal Water Treatment Facility in King County, WA, USA. *E. coli* was added to the wastewater to give a final concentration of 10^7^ CFU/100 mL. The running of the experiment followed the same protocol as above. Samples were collected in a sterile sample bottle and sodium thiosulfate was added to halt the chemical reaction. All samples were enumerated for bacteria following the Standard Methods for the Examination of Water and Wastewater: 9215 Heterotrophic Plate Count (2000) guidelines^29^.

### NSF P231 Challenge Water General Test Water 3 (GTW3) water preparation

1 L of GTW3 was prepared by adding sodium chloride (105 g), humic acid (28.57 mg), sodium bicarbonate (0.1 g), ISO test dust (0.1 g) and *R. terrigena* (10^7^ CFU/100 mL) resulting in a pH of 9.0 ± 0.5. An influent sample of GTW3 Challenge Water was collected in a sterile bottle and set aside as a control.

## Author Contributions

S.A. and S.F.T. drafted the manuscript text and prepared all figures. All authors reviewed the manuscript. P.M., S.F.T. and M.J.A. designed and constructed the swirl flow bioreactor. S.A., S.F.T., R.B., A.O., A.B., R.P. and M.J.A. undertook the field trials in Pune, India. Z.D.G., M.K.S. and A.W. undertook the trials in Seattle, USA. P.G. and M.J.A. conceived the work.

## Additional Information

**How to cite this article**: Atkinson, S. *et al.* Swirl Flow Bioreactor coupled with Cu-alginate beads: A system for the eradication of Coliform and *Escherichia coli* from biological effluents.. *Sci. Rep.* 5, 9461; DOI:10.1038/srep09461 (2015).

## Figures and Tables

**Table 1:** The geographical and physicochemical characteristics of the effluents tested during this study

Geographical Location	Effluent Types	Physical characteristics				
		pH	Chemical Oxygen Demand (COD) (mg/L)	Total Suspended Solids (TSS) (mg/L)	General Appearance	
Hinjewadi Pune	Discharged effluent	7.2	55	9	Very low solid content, transparent.	
Hinjewadi Pune	Secondary clarifier effluent	7.1	141	84	Some solid content, some light brown colouring.	
Hinjewadi Pune	Aeration tanks effluent	7.5	309	132	High solid content, dark brown in colour.	
Seattle Washington	NSF P231 Challenge Water	9.0	44.8	100	Opaque, dark brown, 56 NTU (<5 micron particle size).	
Seattle Washington	Secondary effluent	7.7	1342	4600	Some solid content, light brown in colour.	
Seattle Washington	Septic tank effluent	10.1	11,040	251,950	Very high solid content, very dark brown, thick.	

**Table 2:** The bacterial reduction obtained during treatment of differing effluent types from the Hinjewadi and Seattle sites, with the swirl flow bioreactor containing Cu-alginate beads. Beads were added at a concentration of 53.85 g/L. Each trial is expressed as the mean of three technical replicates

Hinjewadi	Trial	Colony forming units (CFU)/mL	0 mins	10 mins	% reduction in CFU	Log removal value (LRV) (standard deviation)
Discharged effluent	Control	Coliform	27226	14832	45.52	0.26 (±0.13)
		*E. coli*	1434	932	33.54	0.18 (±0.09)
	1	Coliform	1390	0	100	3.12 (±1.57)
		*E. coli*	207	0	100	2.31 (±1.16)
	2	Coliform	8033	0	100	3.90 (±1.95)
		*E. coli*	1243	32	97.89	1.59 (±0.79)
Secondary clarifier effluent	control	Coliform	9844	25601	−146.27	−0.41(±0.21)
		*E. coli*	11308	6369	44.92	0.249 (±0.12)
	1	Coliform	58902	4	99.997	4.16 (±2.04)
		*E. coli*	2994	119	95.82	1.40 (±0.70)
	2	Coliform	38545	1	99.99	4.59 (±2.14)
		*E. coli*	4973	32	99.34	2.19 (±1.09)
Aeration tank effluent	1	Coliform	74108	0	100	4.86 (±2.43)
		*E. coli*	44885	1297	97.11	1.54 (±0.77)

Seattle Washington	Trial	Colony forming units (CFU)/mL	0 mins	10 mins	% reduction in CFU	Log removal value (LRV) (standard deviation)
NSF P231 challenge water	1	*Raoultella terrigena*	470000	0	100	5.67 (±2.84)
	2		470000	0	100	5.67 (±2.84)
Secondary effluent		*E. coli*	110000	0	100	5.04 (±2.52)
Septic tank effluent	1	*E. coli*	150000	800	99.47	2.27 (±1.14)
	2		480000	300000	37.55	0.20 (±0.10)
